# PROK1 Expression Is Associated With Prognosis and May Influence Response to Antiandrogen Therapy in Prostate Cancer

**DOI:** 10.1155/proc/2183439

**Published:** 2026-09-29

**Authors:** Ding-jin Lu, Hai-qi Liang, Yu-jian Li, Jia-yin Yu, Li-wei Wei, Qi-zhou Mo, Jia-wen Zhao, Ren-jun Nong, Yao-yu Wang, Xiao-ping Zhang, Min Qin, Ji-wen Cheng

**Affiliations:** ^1^ Department of Urology, The First Affiliated Hospital of Guangxi Medical University, Nanning, Guangxi, China, gxmu.edu.cn; ^2^ Department of Urology, Beihai People’s Hospital, Beihai, Guangxi, China; ^3^ Department of Urology, Liuzhou Traditional Chinese Medical Hospital, Liuzhou, Guangxi, China, liuzhou.gov.cn; ^4^ Human Sperm Bank, The First Affiliated Hospital of Guangxi Medical University, Nanning, Guangxi, China, gxmu.edu.cn

**Keywords:** antiandrogen therapy, cell communication, mesenchymal stromal cells, prostate cancer, virtual knockout

## Abstract

**Background:**

Previous studies have found that PROK1 is involved in the progression of tumors. This study aims to investigate the association of PROK1 with prognosis and its potential relationship with the efficacy of antiandrogen therapy in prostate cancer and to explore the underlying mechanisms.

**Methods:**

A total of 498 and 1249 prostate cancer samples were obtained from the TCGA and GEO databases, respectively. First, the expression of PROK1 in prostate cancer was analyzed in the bulk RNA sequencing dataset, and the prognostic value of PROK1 was explored. Subsequently, pseudotime analysis and virtual knockout were conducted in the single‐cell RNA sequencing dataset to investigate its biological function in tumor progression. Further cell communication analysis revealed the interactions between cells. Additionally, the predictive function and changes of PROK1 expression in antiandrogen therapy were explored through the GDSC database and in vitro experiments.

**Results:**

PROK1 was downregulated in prostate cancer and decreased with advancing TNM stage. Meta‐analysis also showed lower PROK1 expression in tumor and metastatic prostate cancer compared to controls. Single‐cell RNA sequencing analysis suggested that PROK1 was specifically highly expressed in mesenchymal stem cells (MSC)–like stromal cells. Virtual knockout of the PROK1 perturbation analysis revealed that the differentially expressed genes were enriched in insulin‐like growth factor receptor signaling pathway. Moreover, PROK1‐positive stromal cells had stronger IGFBP signal communication with prostate cancer cells. Additionally, PROK1 and IGFBP7 showed spatial expression colocalization. High PROK1 expression in prostate cancer was associated with higher sensitivity to bicalutamide. However, PROK1 expression decreases after antiandrogen therapy.

**Conclusion:**

Low PROK1 expression is associated with worse prognosis and may correlate with reduced antiandrogen therapy efficacy. Downregulation of PROK1 after antiandrogen therapy may be related to the treatment resistance.

## 1. Introduction

According to the latest data from Global Cancer Statistics, the incidence rate of prostate cancer worldwide is 29.4 per 100,000 people, ranking second among all male malignant tumors globally [1]. For metastatic hormone‐sensitive prostate cancer (mHSPC), antiandrogen therapy is the cornerstone of treatment, aiming to lower the level of androgens in the body to inhibit the growth of cancer cells [2]. Once the disease progresses to metastatic castration‐resistant prostate cancer (mCRPC), tumor growth or spread continues even when androgen levels are extremely low, thus reducing the efficacy of antiandrogen therapy [3].

PROK1 (Prokineticin 1) is a protein‐coding gene. PROK1 is also known as endocrine gland–derived vascular endothelial growth factor (EG‐VEGF) [4]. The protein encoded by this gene can induce the proliferation, migration, and formation of fenestrated membranes (i.e., membrane discontinuities) in capillary endothelial cells from endocrine glands, which is a process that induces cell growth [5, 6]. In our previous studies, it was found that PROK1 is lowly expressed in prostate cancer, and its expression level is correlated with the prostate‐specific antigen (PSA) as a monitoring indicator of prostate cancer [7]. As a vascular growth factor, PROK1 was found in mouse experiments to be inhibited by anti‐PROK1 monoclonal antibodies in angiogenesis and tumor growth [8]. In neuroblastoma, PROK1 activates the AKT pathway to induce tumor cell proliferation [4]. In pancreatic cancer, PROK1 induces antitumor effects through the PI3K/AKT/mTOR pathway and related molecules [9]. Recently, Qin et al. also identified PROK1 using machine learning methods as a predictor of prostate cancer diagnosis and prognosis [10]. However, the underlying mechanism by which PROK1 expression affects the prognosis of prostate cancer remains unclear.

This study employed bioinformatics methods and in vitro experiment to identify the expression level of PROK1 in prostate cancer and its prognostic role. Further exploration was conducted on the biological functions of PROK1 in prostate cancer and its impact on the phenotype of cancer cells. Additionally, the correlation between PROK1 expression and the efficacy of bicalutamide was analyzed using the Cancer Drug Sensitivity Genomics (GDSC) database [11], and the expression changes of PROK1 and its related genes were verified using prostate cancer cell lines treated with bicalutamide and enzalutamide.

## 2. Materials and Methods

### 2.1. Data Source

The bulk RNA sequencing data of prostate cancer from The Cancer Genome Atlas (TCGA) and the clinical information were obtained from the Genomic Data Commons (GDC) Data Portal. This included 498 cases of prostate cancer and 53 cases of adjacent normal prostate tissue samples. Subsequently, bulk RNA sequencing data of 99 benign prostatic hyperplasia tissue samples from the Genotype‐Tissue Expression (GTEx) database were downloaded as normal controls. Bulk RNA sequencing data of 1937 tumors and 493 normal control samples from the Gene Expression Omnibus (GEO) database were also downloaded. Among them, 215 cases were metastatic prostate tumors. To avoid batch effects, raw count data from TCGA and GTEx were obtained, and low‐expressed genes with fewer than 10 counts in more than 50% of samples were filtered out. The remaining counts were then normalized using the median‐of‐ratios function in the DESeq2 R package. To correct for batch effects across datasets, the normalized log2‐transformed counts were adjusted using the ComBat function from the sva *R* package, with data source as a batch covariate. Single‐cell RNA sequencing data of seven prostate tumors were obtained from the GSE185344 dataset [12]. Additionally, spatial transcriptomic data of five untreated prostate tumors and five CRPC cases were acquired from the GSE278936 dataset [13].

### 2.2. Survival Analysis

The Kaplan–Meier curves were plotted using the survival *R* package, with the expression levels as the dividing criterion. The differences in overall survival (OS), progression‐free interval (PFI), disease‐specific survival (DSS), and disease‐free interval (DFI) between high‐ and low‐expression groups of PROK1 in prostate cancer patients were analyzed. Univariate and multivariate Cox regression analyses were performed to determine whether age, TNM stage, PSA level, and PROK1 expression were prognostic factors for OS in prostate cancer. Further, a nomogram for predicting OS prognosis in prostate cancer based on the above factors was plotted using the rms *R* package, and a calibration curve was generated to validate the nomogram’s performance.

### 2.3. Meta‐Analysis

The bulk RNA sequencing data from TCGA and GEO datasets (Supporting Table 1) were analyzed for expression level differences using the meta *R* package. Standardized mean difference (SMD) was calculated to compare PROK1 expression between prostate tumor and normal prostate tissues and between metastatic and nonmetastatic prostate cancer. Subsequently, a meta‐analysis of SMD values was performed to calculate the pooled estimate and assess the overall difference. Moreover, based on the sensitivity and specificity of PROK1 in different datasets for differentiating prostate tumors and metastatic prostate cancer, a summary receiver operating characteristic (SROC) curve was drawn, and the combined sensitivity and specificity were calculated.

### 2.4. Differential Expression Analysis

In the TCGA prostate cancer dataset, tumor samples were divided into high‐ and low‐expression groups based on the median expression level of PROK1. Differential gene expression analysis was performed using the edgeR *R* package for the two groups. The differentially expressed genes (DEGs) were selected based on an absolute value of log2 fold‐change greater than 1 and *p* value less than 0.05. Taking gene expression in the PROK1‐low group as baseline, DEGs with higher expression in the PROK1‐high group were defined as upregulated, those with lower expression as downregulated, and the remaining genes as nonsignificant.

### 2.5. Biological Function Enrichment Analysis

Based on the Gene Ontology (GO) database, biological function enrichment analysis was performed using the clusterProfiler *R* package. The GO functional enrichment analysis defined three different dimension core terms for DEGs: cellular component (CC), molecular function (MF), and biological process (BP). Further, the gene set variation analysis (GSVA) was used to calculate the characteristic GO term scores for all TCGA prostate cancer samples, and the correlation between PROK1 expression level and the characteristic GO term scores was evaluated through the Pearson correlation analysis.

### 2.6. Single‐Cell Sequencing Analysis

The data processing and quality control procedures of the single‐cell sequencing dataset GSE185344 were performed according to the original study [12]. Using the Seurat software package (version 4.0), the single‐cell RNA sequencing data of seven tumor samples were integrated and analyzed. First, quality control was performed based on the number of genes detected per cell (nFeature_RNA), total UMI count (nCount_RNA), and mitochondrial gene percentage (percent.mt). Low‐quality cells with gene counts < 200 or > 5000, UMI counts < 1000, or mitochondrial percentage > 20% were excluded. Subsequently, the Harmony algorithm was used to correct batch effects and merge the data of seven samples. After log normalization of the merged expression matrix, highly variable genes were selected for principal component analysis (PCA). Based on the first 30 principal components of PCA, the UMAP algorithm was used for nonlinear dimensionality reduction, and the Louvain clustering algorithm based on graph was used to identify cell clusters. Finally, cell‐type annotations for each cluster were performed by combining known cell‐type marker genes (Table 1) with the SingleR database.

**TABLE 1:** Gene markers of all cell types in single‐cell RNA sequencing analysis.

Cell	Markers
Epithelial cells	EPCAM, KRT8, KRT18, KRT19, CDH1
Endothelial cells	PECAM1, CDH5, VWF, KDR, CLDN5
Fibroblasts	COL1A1, COL1A2, DCN, PDGFRA, FAP
Stromal cells	PDGFRA, PDGFRB, COL1A1, VIM, ACTA2
MSC‐like stromal cells	PDGFRA, CD44, THY1, ENG, NT5E, CD29
CAFs	FAP, ACTA2, PDGFRB, POSTN, S100A4, TGFB1
Smooth muscle cells	ACTA2, TAGLN, CNN1, MYH11, DES
Pericytes	RGS5, MCAM, PDGFRB, CSPG4, KCNJ8, ABCC9
T cells	CD3D, CD3E, CD2, CD7, PTPRC, CD4, CD8A
B cells	MS4A1, CD79A, CD19, PAX5, IGHM
Macrophages	CD68, CD163, CSF1R, ITGAM, MSR1, MRC1
NK cells	NCAM1, KLRD1, KIR family, NKG7, FCGR3A
Mast cells	KIT, TPSAB1, CPA3, FCER1A, MS4A2
Neurons	RBFOX3, MAP2, TUBB3, SYN1, SNAP25
Ciliated cells	FOXJ1, DNAH5, DNAI1, TUBB4B, SNTN, CCDC78

### 2.7. Identification of Stromal Cell Subpopulations and Pseudotime Analysis

To deeply analyze the heterogeneity of stromal cells, all clusters annotated as stromal cells from the single‐cell dataset were extracted and subjected to subpopulation reclustering analysis. Firstly, the stromal cell subset was renormalized and high‐variable genes were screened. Dimensionality reduction was performed using PCA, and clustering based on the first 20 principal components was performed to identify distinct stromal cell subclusters. Subsequently, to explore potential differentiation trajectories among these subclusters, pseudotime analysis was performed using Monocle 3 (version 1.0). The reclustered stromal cell subclusters were used as input, a principal graph was constructed using the reduced‐dimensional space, pseudotime values were calculated for each cell, and DEGs changing along pseudotime were identified to infer developmental hierarchies and differentiation directions among subclusters.

### 2.8. Virtual Gene Knockout

To explore the function of the PROK1 gene in stromal cells, this study used scTenifoldKnk for virtual gene knockout simulation analysis. scTenifoldKnk is a machine learning–based analysis framework that can construct gene regulatory networks from single‐cell transcriptome data and predict the gene knockout effect by computer simulation of the target genes in the network [14]. The expression matrix of the annotated stromal cell subpopulation was used as input, and a single‐cell gene regulatory network (scGRN) was constructed with default parameters. Then, PROK1 was removed from the wild‐type scGRN adjacency matrix and the knocked‐out scGRN was compared with the wild‐type scGRN to identify differentially regulated genes. Differentially regulated genes were selected using an adjusted *p*‐value < 0.05. By analyzing the global changes in the downstream gene expression network after PROK1 knockout, the potential regulatory function of this gene in stromal cells was evaluated.

### 2.9. Cell Communication Analysis

To explore the mechanism of the effect of PROK1‐positive cells on tumor cells, stromal cell clusters were divided into PROK1‐positive and PROK1‐negative subtypes based on the average expression level. Subsequently, CellChat (version 1.6.1) was used to systematically analyze the ligand–receptor‐mediated cell communication between all‐cell clusters [15]. CellChat integrates the ligand–receptor interaction database and calculates the communication probability for each ligand–receptor pair based on the mass action model and conducts significance assessment using the permutation test (number of permutations = 100). After network construction, outgoing and incoming signaling strengths of each cell population were calculated to quantify their global roles in the signaling communication network. Network topology analysis was used to identify differential signaling pathways and key ligand–receptor pairs between PROK1‐positive and PROK1‐negative stromal cells.

### 2.10. Spatial Transcriptomic Analysis

The spatial transcriptomic analysis was based on the GSE278936 dataset and followed the data processing method of the original study [13]. The analysis was performed in the Python (version 3.9) environment, mainly using Scanpy (version 1.9), Squidpy (version 1.3), and BayesSpace toolkits. Firstly, the data of five treatment naive prostate cancer (TNPC) and CRPC samples were read separately to generate AnnData objects. Then, quality control was performed, removing spots with UMI counts < 500 or > 50,000, fewer than 200 detected genes, and mitochondrial gene proportion > 20%. Subsequently, library size normalization was performed using sc.pp.normalize_total, followed by log transformation using sc.pp.log1p. Highly variable genes were selected using sc.pp.highly_variable_genes. PCA was performed on the integrated expression matrix, and a neighborhood graph was constructed using sc.pp.neighbors based on the first 30 principal components. The Leiden algorithm (resolution 0.6) was used for clustering to identify the spatial domain. Each cluster was annotated with cell types using known cell‐type marker genes. Further, the clustering labels and cell‐type colors were projected onto the original spatial coordinates of the tissue section using Scanpy, generating a spatial distribution map of cell types. Finally, representative feature genes were selected, and spatial heatmaps of their expression abundance on tissue sections were plotted using sc.pl.spatial to reveal the spatial expression patterns of key genes in prostate cancer.

### 2.11. Genomics of Drug Sensitivity in Cancer (GDSC) Analysis

Based on the training dataset of the GDSC database, which includes cell line gene expression profiles and corresponding half‐maximal inhibitory concentration (IC50) values for drugs, this study constructed drug response prediction models using the *R* packages pRRophetic and oncoPredict. The gene expression profiles of the TCGA prostate cancer project were used as the test set, and the predicted IC50 values of each drug in each sample were quantified using the ridge regression algorithm to evaluate the sensitivity of each sample to the selected ADC and chemotherapy drugs. The GSE111177 dataset contains sequencing data of paired tumor samples from 24 prostate cancer patients before and after antiandrogen therapy [16]. The effect of bicalutamide usage on the expression level of PROK1 was further investigated.

### 2.12. Cell Lines and Culture Conditions

The human prostate cancer cell line LNCaP was cultured in RPMI‐1640 medium containing 10% fetal bovine serum, and umbilical cord blood–derived mesenchymal stem cells (MSCs) were cultured in the dedicated mesenchymal stem cell medium (Pricella, CM‐H165) [17]. All cells were grown adherently at 37°C in a 5% CO_2_ atmosphere, with medium changed every 2–3 days. Cells from passages 3–6 were used for experiments. For drug treatments, cells were seeded at a density of 2 × 10^5^ cells/well (LNCaP) or 1 × 10^5^ cells/well (MSCs) in 6‐well plates and allowed to attach overnight. Cells were then treated with bicalutamide and enzalutamide at a range of concentrations (0, 0.1, 1, 10, 50, and 100 μM) for 48 h. All drug‐treated groups were compared with untreated controls under identical culture conditions. Each experiment included three independent biological replicates, with three technical replicates per condition. All cell lines used in this study were authenticated by STR profiling. They were routinely tested for mycoplasma contamination using a PCR‐based assay and were consistently negative throughout the experiments.

### 2.13. siRNA Transfection and Quantitative Real‐Time PCR (qRT‐PCR)

MSCs were seeded in 6‐well plates at a density of 2 × 10^5^ cells per well and cultured until reaching 60%–70% confluence. Cells were then transfected with 50 nM of PROK1‐specific siRNA (si‐PROK1) or 50 nM of negative control siRNA (si‐NC) using Lipofectamine RNAiMAX (Invitrogen, Carlsbad, CA, USA) according to the manufacturer’s instructions. After 48 h of transfection, total RNA was extracted using TRIzol reagent (Invitrogen), and cDNA was synthesized using the PrimeScript RT Reagent Kit (Takara, Kyoto, Japan). qRT‐PCR was performed using TB Green Premix Ex Taq II (Takara) on a LightCycler 480 System (Roche, Basel, Switzerland). The relative expression levels of PROK1, IGFBP7, IGFBP4, and IGFBP6 were calculated using the 2^−^ΔΔCt method, with GAPDH as the internal reference gene. All transfections and qRT‐PCR reactions were performed in three biological replicates, each with three technical replicates. The siRNA and primer sequences are listed in Table 2.

**TABLE 2:** The sequences of siRNA/primer.

siRNA/primer	Type	Sequence
PROK1 siRNA	SS Sequence	AAUUGAUGUUCUUCAAGUCCA
	AS Sequence	GACUUGAAGAACAUCAAUUUU

PROK1 primer	Forward primer	AGGCATCTAAGCAGGCAGTG
	Reverse primer	GCGTTTCCTGAAGAAGGGGA

IGFBP7 primer	Forward primer	AAGAGGCGGAAGGGTAAAGC
	Reverse primer	TGCCCTTATGGGTTGCTAACT

IGFBP4 primer	Forward primer	GCAGAAGCACTTCGCCAAAA
	Reverse primer	GGAGTGTCTCCACATGCCAA

IGFBP6 primer	Forward primer	CTGCAGCAACTCCAGACTGA
	Reverse primer	CGGTAGAAGCCTCGATGGTC

### 2.14. RNA Sequencing

To identify the expression changes of PROK1 and characteristic genes in prostate cancer cells and MSCs stimulated by bicalutamide or enzalutamide, RNA sequencing was performed on the following samples: bicalutamide‐stimulated prostate cancer cells (Bic), enzalutamide‐stimulated prostate cancer cells (Enz), control prostate cancer cells; bicalutamide‐stimulated MSCs (Bic‐MSCs), enzalutamide‐stimulated MSCs (Enz‐MSCs), and control MSCs. Total RNA was extracted from each group using the TRIzol method, and the purity and integrity of RNA were detected using NanoDrop 2000 and Agilent Bioanalyzer 2100. Dual‐end 150 bp (PE150) sequencing was performed on the Illumina NovaSeq 6000 platform. The raw data were quality‐controlled by FastQC and deadapter‐removed and low‐quality reads were filtered using Trimmomatic. Then, they were aligned to the human reference genome (GRCh38) using HISAT2 and quantified by featureCounts.

### 2.15. Statistical Analysis

Statistical analysis was completed using *R* software (version 4.2), Python (version 3.9), and Stata (version 15). Comparisons between two groups were performed using the Wilcoxon rank sum test or independent sample *t*‐test, and comparisons among multiple groups were performed using the Kruskal–Wallis test. The Kaplan–Meier survival analysis was conducted with log‐rank test. In univariate and multivariate Cox regression models, results were expressed as hazard ratios (HRs) and their 95% confidence intervals (CIs). All tests were two‐sided, and *p* < 0.05 was considered statistically significant.

## 3. Result

### 3.1. PROK1 Is Expressed at a Low Level in Prostate Tumors

In the TCGA prostate cancer dataset, the expression level of PROK1 in prostate tumors was lower than in adjacent normal tissues (*p* = 2.1e − 07; Figure 1A). As the *T* stage progressed, the expression level of PROK1 in prostate tumors gradually decreased (*p* = 3.5e − 10; Figure 1B). Moreover, the expression level of PROK1 also decreased with the progression of *N* stage (*p* = 8.9*e* − 09; Figure 1C). The expression level of PROK1 also decreased with the progression of *M* stage (*p* = 0.031; Figure 1D). PROK1 expression in patients with PSA > 4 was also lower than in those with PSA < 4 (*p* = 0.002; Figure 1E). In addition, the expression level of PROK1 also decreased with the increase of the Gleason score (*p* = 2.0*e* − 16; Figure 1F). PROK1 was also found downregulated in prostate tumors in GSE32571, GSE32982, GSE35988, GSE46602, GSE72220, GSE94767, GSE104749, and GSE134051 datasets (*p* < 0.05; Figure 1G–N).

**FIGURE 1:**
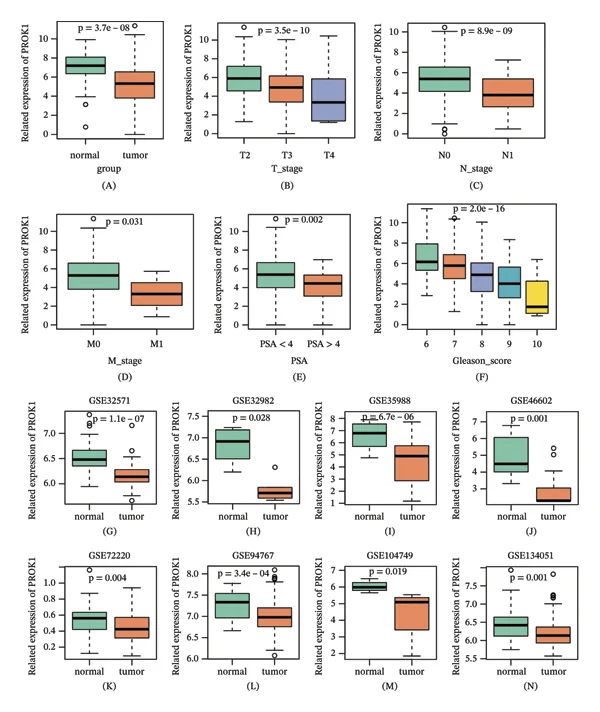
Expression patterns of PROK1 in prostate cancer across TCGA‐PRAD and multiple independent cohorts. (A) Comparison of PROK1 between normal and tumors. (B) Comparison of PROK1 between different *T* stages. (C) Comparison of PROK1 between N0 and N1 stages. (D) Comparison of PROK1 between M0 and M1 stages. (E) Comparison of PROK1 between PSA < 4 and PSA > 4 tumors. (F) Comparison of PROK1 between different Gleason scores. (G–N) PROK1 expression in GSE32571, GSE32982, GSE35988, GSE46602, GSE72220, GSE94767, GSE104749, and GSE134051 datasets.

### 3.2. Low Expression of PROK1 Is Associated With Poor Prognosis

In the TCGA prostate cancer dataset, patients with a low expression of PROK1 in prostate cancer had poorer OS, PFI, DSS, and DFI (Figure 2A–D). In the univariate Cox regression analysis, *N* stage, *M* stage, PSA value, and PROK1 expression level were all risk factors for prostate cancer OS (Figure 2E). However, in the further multivariate Cox regression analysis, only the PSA value was an independent risk factor for prostate cancer OS (Figure 2F). A nomogram for predicting the OS of prostate cancer patients was constructed based on age, *T* stage, *N* stage, PSA value, and PROK1 expression level (Figure 2G), and the calibration curve showed good accuracy (Figure 2H).

**FIGURE 2:**
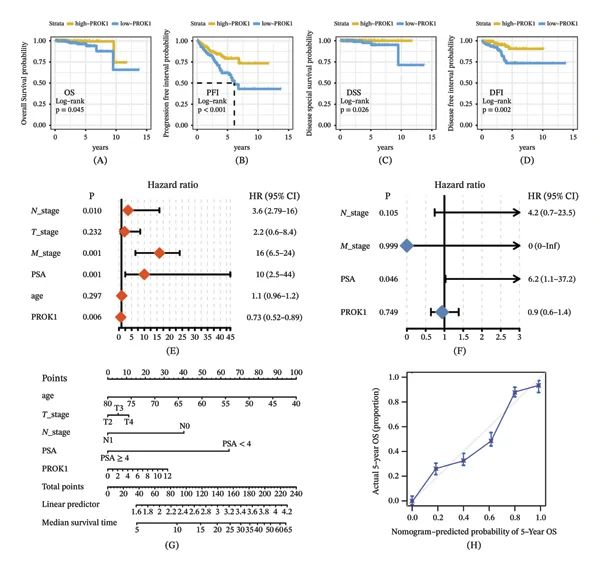
Prognostic value of PROK1 in prostate cancer patients from TCGA‐PRAD. (A–D) Kaplan–Meier survival curves comparing high‐ and low‐PROK1 expression groups for (A) overall survival (OS), (B) progression‐free interval (PFI), (C) disease‐specific survival (DSS), and (D) disease‐free interval (DFI). (E) Forest plot showing the results of univariate Cox regression analysis for PROK1 and other clinical variables (age, *T* stage, *N* stage, *M* stage, Gleason score, PSA level) in relation to OS. Hazard ratios (HRs) with 95% confidence intervals (CIs) are shown. (F) Forest plot of multivariate Cox regression analysis incorporating all significant variables from univariate analysis. (G) Nomogram integrating PROK1 expression and clinical parameters for predicting median OS. (H) Calibration curve evaluating the predictive accuracy of the nomogram for 5‐year OS.

### 3.3. PROK1 Is Expressed at a Lower Level in Metastatic Prostate Cancer

Meta‐analysis integrating 17 datasets comprising 1372 prostate tumors and 493 benign prostate tissues found reduced PROK1 expression in prostate tumors (Figure 3A). Integration of 5 datasets containing 215 metastatic and 350 nonmetastatic prostate cancer samples also found reduced PROK1 expression in metastatic prostate tumors (Figure 3B). The SROC curve integrated the ROC curves from all datasets to differentiate prostate tumors and benign prostate tissues. The AUC of the integrated data was 0.80 (95% CI 0.76–0.83), and the integrated sensitivity and specificity were 0.77 (95% CI 0.65–0.85) and 0.70 (95% CI 0.56–0.81), respectively (Figure 3C). The SROC curve integrating the expression level of PROK1 for differentiating metastatic prostate cancer and nonmetastatic prostate cancer showed that the AUC was 0.87 (95% CI 0.84–0.90), and the integrated sensitivity and specificity were 0.75 (95% CI 0.60–0.85) and 0.90 (95% CI 0.72–0.97), respectively (Figure 3D).

**FIGURE 3:**
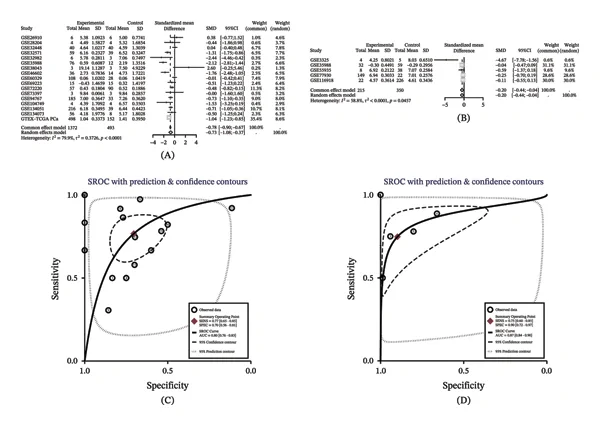
Meta‐analysis of PROK1 expression and its diagnostic value in prostate cancer. (A) Forest plot of the standardized mean difference (SMD) for PROK1 expression between prostate cancer and benign prostatic tissues across multiple independent cohorts. A negative SMD indicates lower PROK1 expression in tumors. (B) Forest plot of SMD for PROK1 expression between nonmetastatic and metastatic prostate cancers, showing lower PROK1 levels in metastatic lesions. (C) Summary receiver operating characteristic (SROC) curve evaluating the diagnostic performance of PROK1 for distinguishing prostate cancer from benign prostatic tissue. The area under the curve (AUC) and 95% CI are reported. (D) SROC curve assessing the ability of PROK1 to discriminate metastatic from nonmetastatic prostate cancer.

### 3.4. The Biological Function of PROK1 Is Related to the Hormone Metabolism Process

Differential gene expression analysis identified 9 upregulated and 20 downregulated DEGs between PROK1‐high and PROK1‐low tumors in TCGA prostate cancer (Figure 4A). The PROK1‐related DEGs were enriched in cAMP‐mediated signal positive regulation, hormone metabolism pathways, adrenergic receptor signaling pathways, 5‐hydroxytryptamine receptor signaling pathways, fibroblast growth factor receptor signaling pathways, and PI3K/PKB signal transduction (Figure 4B). GSVA analysis showed that the expression level of PROK1 was strongly correlated with the activity of hormone metabolism pathways, adrenergic receptor signaling pathways, and 5‐hydroxytryptamine receptor signaling pathways (Figure 4C). PROK1 expression was positively correlated with the hormone metabolism–related gene RPE65 but negatively correlated with AFP and UGT2B11 expression (Figure 4D).

**FIGURE 4:**
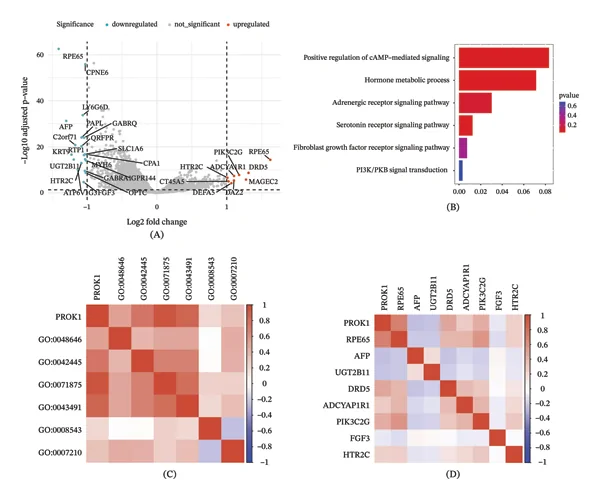
Transcriptomic landscape associated with PROK1 expression in prostate cancer. (A) Volcano plot of differentially expressed genes (DEGs) between PROK1‐high and PROK1‐low expression groups. Significantly upregulated genes are shown in red, downregulated genes in blue, and nonsignificant genes in gray. (B) Gene Ontology (GO) enrichment analysis of the DEGs, showing the top 10 most significantly enriched biological processes and pathways. (C) Pearson correlation analysis between PROK1 expression levels and the enrichment scores of pathways identified in (B). (D) Pearson correlation analysis between PROK1 expression and the average expression of signature genes from key enriched pathways. Positive correlations indicate concordant regulation.

### 3.5. PROK1 Is Mainly Expressed in Prostate Cancer Mesenchymal Stem Cells

Single‐cell sequencing analysis identified 11 cell clusters in the tumor microenvironment: endothelial cells, T cells, epithelial cells, macrophages, NK cells, stromal cells, B cells, mast cells, fibroblasts, neurons, and ciliated cells (Figure 5A). Among them, PROK1 was mainly expressed in stromal cells (Figure 5B). Further, the stromal cell clusters were divided into four subclusters: MSC‐like stromal cells, cancer‐associated fibroblasts (CAFs), smooth muscle cells (SMCs), and pericytes (Figure 5C). Through pseudotime analysis, the differentiation trajectory of stromal cell clusters was inferred and visualized, and PROK1 was mainly highly expressed in the early differentiation stage of stromal cells, which was identified as MSC‐like stromal cells (Figure 5D–F). The cell cluster types and the subclusters of stromal cells were verified through heatmaps of marker gene expression (Figure 5G,H).

**FIGURE 5:**
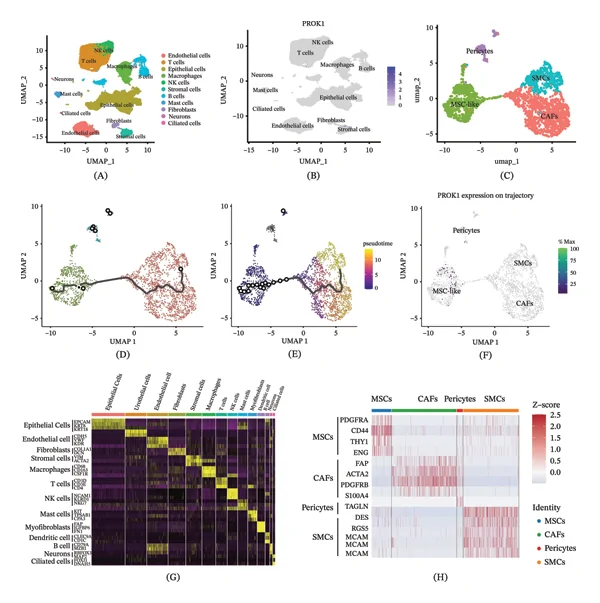
Single‐cell RNA sequencing reveals stromal‐restricted expression of PROK1. (A) UMAP dimensional reduction plot of all cells from the GSE185344 scRNA‐seq dataset, colored by cell‐type annotation. (B) UMAP plot displaying PROK1 expression levels across all cells, with color intensity reflecting log‐normalized expression. (C) UMAP plot of the stromal cell subpopulation, reclustered into distinct stromal subsets. (D, E) Pseudotime trajectory analysis of stromal cells showing the developmental progression (D) and branch points (E), inferred using Monocle 3. (F) UMAP plot displaying PROK1 expression specifically within the stromal cell population. (G) Heatmap showing the expression of canonical cell–type marker genes across all major cell clusters identified in (A). (H) Heatmap displaying the expression of marker genes across stromal subclusters, demonstrating the molecular heterogeneity within the stromal compartment.

### 3.6. PROK1 Participates in the Regulation of IGF Receptor Signaling

Virtual gene knockout experiments showed that genes whose expression levels changed significantly after PROK1 gene knockout included genes related to the insulin‐like growth factor (IGF) receptor signaling pathway, such as IGFBP7, IGFBP4, IGFBP2, IGFBP6, and IGFBP5 (Figure 6A–C). These DEGs were enriched in steroid hormone response, IGF receptor signaling pathways, and IGF/IGF receptor association biological functions (Figure 6D). IGFBP2 was simultaneously a key gene in both steroid hormone response and IGF receptor signaling pathways, and IGFBP7 was a key gene in both steroid hormone response and IGF/insulin‐like growth factor binding molecular functions (Figure 6E). Based on the results of virtual gene knockout, a protein interaction network was constructed and showed complex interactions between PROK1 and IGF receptor signaling pathway–related genes (Figure 6F).

**FIGURE 6:**
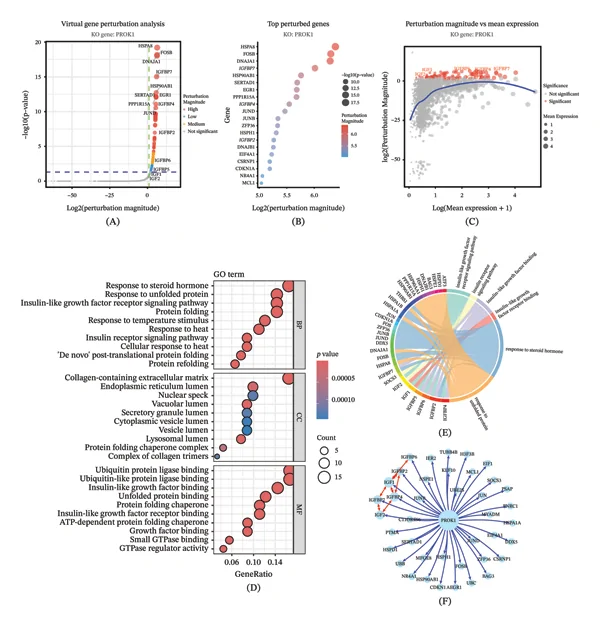
Virtual knockout analysis of PROK1 and its downstream molecular consequences. (A) Volcano plot of differentially expressed genes after in silico PROK1 knockout, comparing perturbed vs. control states. (B) Bubble plot highlighting the top DEGs with their log_2_FC and statistical significance. (C) Perturbation magnitude plotted against the mean expression levels for all DEGs. (D) Gene function enrichment analysis of genes affected by PROK1 knockout, identifying key biological pathways and molecular functions, with circle size representing gene count and color indicating significance level. (E) Enrichment of DEGs in selected oncogenic and signaling pathways. (F) Protein–protein interaction (PPI) network constructed from PROK1 and its top correlated DEGs.

### 3.7. PROK1‐Positive Stromal Cells Enhance IGF Receptor Signaling Communication With Prostate Cancer Cells

Cell communication analysis showed that PROK1‐positive stromal cells had more communication numbers between cell clusters than negative cells (Figure 7A), but there was no significant difference in the intensity (Figure 7B). The heat map of communication numbers and intensity again verified the above findings (Figure 7C,D). Further analysis of all intercell communication signals revealed that the signal strength of IGF and IGFBP in the signals emitted by PROK1‐positive stromal cells was significantly stronger than that of PROK1‐negative cells (Figure 7E). The receiving signals of epithelial cells were also stronger for IGFBP (Figure 7F). In the separate analysis of IGFBP signal communication, PROK1‐positive stromal cells were the main signal emitters, while epithelial cells were the main receivers (Figure 7G). In the interaction molecule pairs between stromal cells and epithelial cells, IGFBP3‐TMEM219 and IGF2‐IGF2R were significantly enhanced in PROK1‐positive stromal cells compared to negative cells (Figure 7H). The strength of the IGFBP signaling pathway network diagram again verified the strong communication between PROK1‐positive stromal cells and epithelial cells (Figure 7I).

**FIGURE 7:**
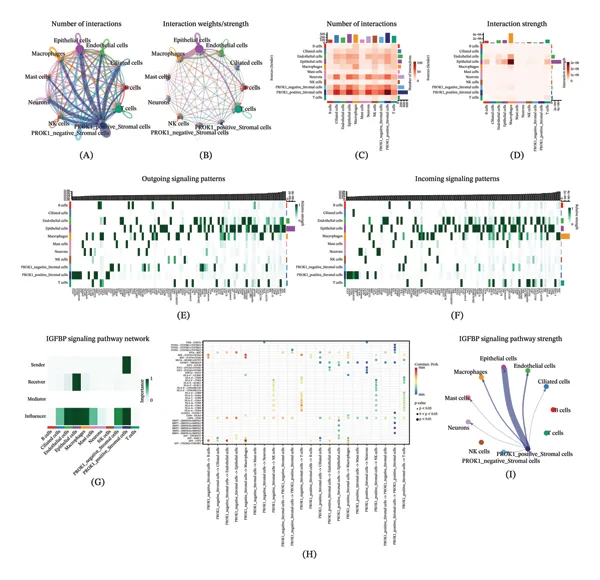
Cell–cell communication network analysis of the prostate cancer microenvironment. (A) Total number of inferred cell–cell interactions between each pair of cell types, computed using CellChat. (B) Total interaction strength (sum of communication probabilities) between each pair of cell types. (C) Heatmap showing the number of interactions between all cell types. (D) Heatmap showing the interaction strength between all cell types. (E, F) Outgoing (E) and incoming (F) signaling strengths for each cell type, indicating the relative contribution of each cell type as a signal sender or receiver. (G) Relative contribution of each cell type to the IGFBP signaling pathway. (H) Interaction strength of ligand–receptor pairs involved in IGFBP signaling between cell types. (I) Comparison of IGFBP signaling communication strength between PROK1‐positive stromal cells and PROK1‐negative stromal cells with other cell types, demonstrating the role of PROK1‐positive stromal cells as the primary source of IGFBP signals.

### 3.8. PROK1 and IGFBP7 Have Spatial Colocalization

Ten cell clusters were identified in the spatial transcriptome data set of TNPC samples: epithelial cells, B cells, endothelial cells, fibroblasts, mast cells, myeloid cells, neurons, MSC‐like stromal cells, and T cells (Figure 8A). PROK1 was coexpressed with IGFBP7, IGFBP4, and IGFBP6, with the highest expression in MSC‐like stromal cells (Figure 8B–E). The Leiden algorithm was used to construct spatial clustering based on gene expression profiles, and 27 cell clusters were divided on the in situ images of five tumor samples (Figure 8F–J). Each spot on the tissue section in situ images was manually annotated based on the marker genes (Figure 8K–O). Spatial gene expression maps of IGFBP7 and PROK1 showed their colocalization in space (Figure 8P–Y). The above 10 cell types were also identified in the five spatial transcriptome datasets of CRPC samples (Figure 9A). In CRPC, PROK1 was coexpressed with IGFBP7, IGFBP4, and IGFBP6 in MSC‐like stromal cells (Figure 9B–E). The tissue sections were similarly annotated into 10 cell types (Figure 9F–O); PROK1 and IGFBP7 again exhibited spatial colocalization (Figure 9P–Y).

**FIGURE 8:**
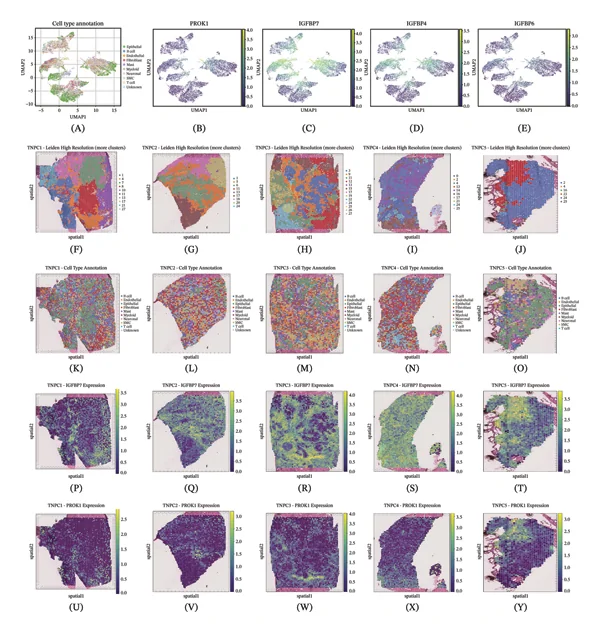
Spatial transcriptomic profiling of TNPC. (A) UMAP dimensional reduction clustering plot of five TNPC tissue sections. (B–E) UMAP plots showing the expression patterns of PROK1, IGFBP7, IGFBP4, and IGFBP6 across TNPC tissues. (F–J) Leiden algorithm–based clustering on tissue sections from five TNPC cases, with different colors representing distinct spatial clusters. (K–O) Cell‐type annotation mapped onto tissue sections, identifying epithelial, stromal, immune, and other microenvironmental compartments. (P–T) Spatial distribution of IGFBP7 expression on representative tissue sections, with color intensity indicating relative expression levels. (U–Y) Spatial distribution of PROK1 expression on representative tissue sections.

**FIGURE 9:**
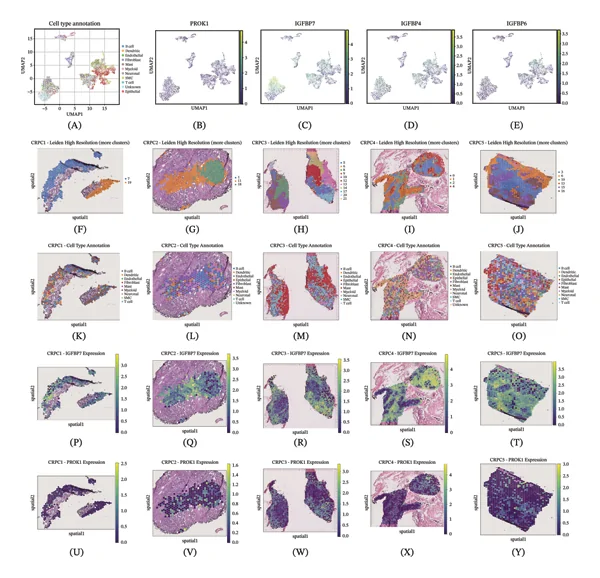
Spatial transcriptomic profiling of CRPC. (A) UMAP dimensional reduction clustering plot of five CRPC tissue sections. (B–E) UMAP plots showing the expression patterns of PROK1, IGFBP7, IGFBP4, and IGFBP6 across CRPC tissues. (F–J) Leiden algorithm–based clustering on tissue sections from five CRPC cases, with different colors representing distinct spatial clusters. (K–O) Cell‐type annotation mapped onto tissue sections, identifying epithelial, stromal, immune, and other microenvironmental compartments. (P–T) Spatial distribution of IGFBP7 expression on representative tissue sections, with color intensity indicating relative expression levels. (U–Y) Spatial distribution of PROK1 expression on representative tissue sections.

### 3.9. High PROK1 Expression Is Associated With Higher Sensitivity to Bicalutamide in Prostate Cancer

Using the GDSC database, analysis of drug sensitivity in TCGA prostate cancer showed that the IC50 values for androgen receptor (AR) pathway inhibitors bicalutamide and AZD3514 and the anti‐EGFR monoclonal antibody cetuximab were lower in the PROK1‐high group compared to that in the PROK1‐low group, indicating higher drug sensitivity (Figure 10A–C). Furthermore, in the dataset GSE111177, the expression level of PROK1 after bicalutamide/enzalutamide treatment was lower than that before treatment (Figure 10D). In our in‐house data, PROK1 expression in both prostate cancer cells and MSCs treated with bicalutamide/enzalutamide was reduced compared to control groups (Figure 10E). Moreover, IGFBP7 expression was also reduced in MSCs treated with bicalutamide/enzalutamide (Figure 10F). IGFBP4 and IGFBP6 expressions were reduced in bicalutamide‐treated MSCs (Figure 10G,H). However, due to limited sample size, these differences were not statistically significant. In the qRT‐PCR analysis, the PROK1 expression was significantly decreased in PROK1‐knockdown MSCs, along with a significant reduction in the expression levels of IGFBP7, IGFBP4, and IGFBP6 (Figure 10I–L).

**FIGURE 10:**
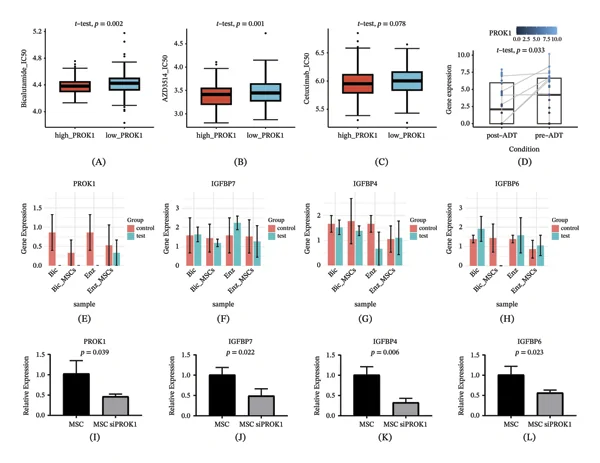
PROK1 expression is associated with sensitivity to AR pathway inhibitors and regulates IGFBP expression. (A) IC50 prediction for bicalutamide treatment. (B) IC50 prediction for AZD3514 treatment. (C) IC50 prediction for cetuximab treatment. (D) PROK1 expression levels before and after antiandrogen therapy in the GSE111177 dataset. (E) PROK1 expression levels in tumor cells of the treatment group versus the control group. (F) IGFBP7 expression levels in tumor cells of the treatment group versus the control group. (G) IGFBP4 expression levels in tumor cells of the treatment group versus the control group. (H) IGFBP6 expression levels in tumor cells of the treatment group versus the control group. (I–L) Expression of PROK1, IGFBP7, IGFBP4, and IGFBP6 in PROK1‐knockdown MSCs.

## 4. Discussion

By integrating multi‐omics data such as bulk RNA sequencing, single‐cell RNA sequencing, and spatial transcriptomics, this study systematically revealed the expression characteristics, cell origin, and prognostic value of PROK1 in prostate cancer and preliminarily elucidated its potential mechanism of influencing the tumor microenvironment through interaction with the IGF signaling pathway. We found that PROK1 was expressed at a lower level in prostate tumors compared to that in benign prostate tissues, and its low expression was associated with poor OS prognosis in prostate cancer patients.

Early studies were mainly based on immunohistochemistry (IHC) and Western blot, reporting that PROK1 was upregulated in prostate cancer [18, 19]. These findings mainly came from analyses focused on epithelial cells. However, recent large‐scale transcriptome studies based on bulk RNA‐seq revealed that PROK1 was significantly downregulated in prostate cancer compared to that in normal tissues [7]. More importantly, functional studies have shown that inhibiting PROK1 expression can promote the proliferation and invasion of prostate cancer cells, while its overexpression has an anticancer effect [10]. The key factor leading to this apparent contradiction may lie in the cellular environment: our single‐cell and spatial transcriptomic data reveal that PROK1 is mainly expressed in stromal/MSC‐like cells, rather than in epithelial tumor cells. Previous studies on the upregulation of PROK1 in cancer mostly focused on the epithelial components [18], while overall tissue‐level analyses including both stroma and epithelial components may present different expression patterns depending on the relative composition of these components.

Gene biological function enrichment analysis suggested that the expression of the PROK1 gene was related to the hormone metabolism process. Among them, the expression of PROK1 and the related gene RPE65 for hormone metabolism process were positively correlated, while it was negatively correlated with AFP and UGT2B11 expression. MSCs are an important component of the tumor microenvironment and can regulate the proliferation, migration, and treatment resistance of tumor cells through paracrine secretion of various cytokines and growth factors [20]. Single‐cell RNA sequencing analysis revealed that PROK1 was mainly expressed in MSC‐like stromal cells in the prostate cancer tumor microenvironment and was concentrated in the early‐differentiated stromal cell population.

More importantly, virtual gene knockout experiments and cell communication analysis revealed that PROK1 in MSC‐like stromal cells may participate in the stromal–epithelial interaction by regulating genes related to the IGF receptor signaling pathway. Through pseudotime analysis, we found that PROK1 was highly expressed in the early stage of stromal cell differentiation and gradually decreased as the differentiation process progressed. This expression pattern suggests that PROK1 may play a certain role in maintaining the undifferentiated state or early functions of MSCs. Previous studies have reported that PROK1 was upregulated in umbilical cord mesenchymal stem cells, but its association with mesenchymal stem cell differentiation is still unclear [21]. Through single‐cell RNA sequencing analysis, we also found that PROK1 was mainly expressed in prostate tumor MSC–like stromal cells. Virtual gene knockout and cell communication analysis suggested that PROK1‐positive MSC‐like stromal cells were associated with prostate cancer cells through the IGF signaling pathway.

The study by Goto et al. through dual fluorescence IHC experiments found that PROK1 and IGFBP1 were significantly colocalized in stromal cells and glandular epithelial cells [22]. This indicates that they are expressed in the same cell types, providing a spatial basis for their functional interaction. Our spatial transcriptomic analysis suggested that PROK1, along with IGFBP7, IGFBP4, and IGFBP6, showed significant spatial colocalization in the MSC‐like stromal cell regions of tissue sections. This pattern was observed in both TNPC and CRPC samples. This further supports the functional synergy between PROK1 and the members of the IGFBP family.

Ujvari et al. demonstrated that overexpression of PROK1 inhibits cell migration and invasion capabilities. Simultaneously, the level of IGF2 will decrease [23]. IGF‐2 can regulate cell proliferation, differentiation, and metabolism through autocrine and paracrine mechanisms [24]. PROK1 is a key node in the IGF pathway. When PROK1 binds to its receptor PROKR1, it activates the downstream PI3K/AKT/mTOR signaling axis, which is exactly the same as the downstream pathway activated after IGF binding to its receptor [25]. Our research found that the IGFBP signals sent by PROK1‐positive MSC‐like stromal cells to prostate cancer cells were significantly stronger than those from PROK1‐negative cells. Moreover, the interaction molecules between PROK1‐positive MSC‐like stromal cells and prostate cancer cells were significantly associated with IGFBP3‐TMEM219 and IGF2‐IGF2R. This further validates that the expression of PROK1 in MSCs may regulate the intensity of IGFBP signals to prostate cancer cells, thereby controlling the biological characteristics of cancer cells.

Our study also found that prostate cancer patients with high PROK1 expression had significantly higher sensitivity to drugs such as bicalutamide and AZD3514 compared to those with low expression, suggesting that PROK1 serves as a biomarker associated with the efficacy of antiandrogen therapy. The results of in vitro experiments showed that the expression levels of PROK1 in prostate cancer cells and MSCs treated with bicalutamide or enzalutamide both showed a downward trend. The GSE111177 dataset also indicated that the expression of PROK1 decreased after antiandrogen therapy. This result may reveal a feedback regulatory mechanism: AR pathway inhibitors can inhibit PROK1 expression, and patients with high PROK1 expression are more sensitive to AR pathway inhibitors; after long‐term antiandrogen therapy, PROK1 is inhibited, which may contribute to the occurrence of treatment resistance.

This study has some limitations. Firstly, the research is mainly based on bioinformatics analysis of public databases; although it included a large number of samples, it lacked independent prospective clinical cohorts for verification. The downregulation of PROK1 in advanced tumors does not necessarily imply a causal role in driving progression. Secondly, the PROK1‐IGFBP/IGF axis is a potential mechanism requiring further functional validation. Moreover, the association of PROK1 with bicalutamide sensitivity is based on GDSC‐predicted IC50, public datasets, and in vitro RNA‐seq, without direct clinical endpoints. These findings are preliminary and require prospective validation.

## 5. Conclusion

This study reveals that PROK1 is lowly expressed in prostate cancer, and that low expression is associated with poor patient prognosis and increased risk of metastasis. PROK1 is mainly expressed on MSC‐like stromal cells, and PROK1‐positive MSC‐like stromal cells may participate in regulating the sensitivity of the androgen signaling pathway by modulating the expression of IGFBP family members. In vitro experiments suggested that prostate cancer cells with higher PROK1 expression exhibit greater sensitivity to bicalutamide and enzalutamide, and the downregulation of PROK1 after antiandrogen therapy may be associated with the development of treatment resistance. The findings of this study provide new theoretical basis and intervention targets for individualized treatment of prostate cancer and research on antiandrogen therapy resistance.

**Nomenclature**
AUC: area under the curve
CAFs: cancer‐associated fibroblasts
CI: confidence interval
CRPC: castration‐resistant prostate cancer
DFI: disease‐free interval
DEGs: differentially expressed genes
DSS: disease‐specific survival
GDSC: Genomics of Drug Sensitivity in Cancer
GEO: Gene Expression Omnibus
GO: Gene Ontology
GSVA: gene set variation analysis
GTEx: Genotype‐Tissue Expression
HR: hazard ratio
IC50: half‐maximal inhibitory concentration
IGF: insulin‐like growth factor
IHC: immunohistochemistry
HSPC: metastatic hormone‐sensitive prostate cancer
MSCs: mesenchymal stem cells
OS: overall survival
PCA: principal component analysis
PFI: progression‐free interval
PSA: prostate‐specific antigen
SMD: standardized mean difference
SMCs: smooth muscle cells
SROC: summary receiver operating characteristic
TCGA: The Cancer Genome Atlas
TNPC: treatment naive prostate cancer
UMAP: uniform manifold approximation and projection

## Funding

This work was supported by the Guangxi Key Research and Development Program (No. GuikeFN2504240032), the National Natural Science Foundation of China (No. 82160483), the Guangxi Clinical Research Center for Urology and Nephrology (No. GuikeAD20297081), and the Guangxi Science and Technology Base and Talent Project (No. GuikeAD20238090).

## Ethics Statement

Ethical approval was not required for this study in accordance with local guidelines.

## Consent

The authors have nothing to report.

## Conflicts of Interest

The authors declare no conflicts of interest.

## Supporting Information

Additional supporting information can be found online in the Supporting Information section.

## Supporting information


**Supporting Information** Supporting File: Supporting Table 1 The RNA‐seq datasets included in the meta‐analysis.

## Data Availability

The publicly available gene expression data used in the present study can be accessed from the TCGA website (https://portal.gdc.cancer.gov/), GEO database website (https://www.ncbi.nlm.nih.gov/gds), and GDSC website (https://www.cancerrxgene.org).
