# Supplementary material for: PROK1 Expression Is Associated With Prognosis and May Influence Response to Antiandrogen Therapy in Prostate Cancer
Source: Prostate Cancer. 2026 Sep 29;2026:2183439. doi: 10.1155/proc/2183439 (PMC13621831; doi:10.1155/proc/2183439)
Supplement: Supplementary file 1 [file PROC-2026-2183439-s001.docx]

**Supplementary Table 1** The RNA-seq datasets included in the meta-analysis.

| Study | Country | Year | Experimental group | Control group | Reference |
| --- | --- | --- | --- | --- | --- |
| GSE26910 | Italy | 2011 | PCa | Normal | [1] |
| GSE32448 | USA | 2011 | PCa | Normal | [2] |
| GSE32982 | Finland | 2011 | PCa | Normal | [3] |
| GSE38043 | USA | 2012 | PCa | Normal | [4] |
| GSE46602 | Denmark | 2013 | PCa | Normal | [5] |
| GSE69223 | Germany | 2015 | PCa | Normal | [6] |
| GSE104749 | China | 2017 | PCa | Normal | [7] |
| GSE72220 | USA | 2015 | PCa | Normal | [8] |
| GSE94767 | United Kingdom | 2017 | PCa | Normal | [9] |
| GSE28204 | China | 2011 | PCa | Normal | [10] |
| GSE134073 | Germany | 2020 | PCa | Normal | [11] |
| GSE60329 | Italy | 2014 | PCa | Normal | [12] |
| GSE73397 | China | 2015 | PCa | Normal | [13] |
| GSE134051 | Germany | 2019 | PCa | Normal | [14] |
| GSE32571 | Germany | 2011 | PCa | Normal | [15] |
| GTEx-TCGA PCa | USA | 2022 | PCa | Normal | [16] |
| GSE3325 | USA | 2005 | Metastatic PCa | PCa | [17] |
| GSE35988 | USA | 2012 | Metastatic PCa | PCa | [18] |
| GSE55935 | USA | 2015 | Metastatic PCa | PCa | [19] |
| GSE77930 | USA | 2016 | Metastatic PCa | PCa | [20] |
| GSE116918 | United Kingdom | 2018 | Metastatic PCa | PCa | [21] |

PCa, Prostate Cancer

Reference

1.	Sun C, Gu Y, Chen G, Du Y. Bioinformatics Analysis of Stromal Molecular Signatures Associated with Breast and Prostate Cancer. J Comput Biol. 2019; 26: 1130-9.

2.	Lu W, Ding Z. Identification of key genes in prostate cancer gene expression profile by bioinformatics. Andrologia. 2019; 51: e13169.

3.	Vaarala MH, Hirvikoski P, Kauppila S, Paavonen TK. Identification of androgen-regulated genes in human prostate. Mol Med Rep. 2012; 6: 466-72.

4.	Huen NY, Pang AL, Tucker JA, Lee TL, Vergati M, Jochems C, et al. Up-regulation of proliferative and migratory genes in regulatory T cells from patients with metastatic castration-resistant prostate cancer. Int J Cancer. 2013; 133: 373-82.

5.	Liang X, Wang Y, Pei L, Tan X, Dong C. Identification of Prostate Cancer Risk Genetics Biomarkers Based on Intergraded Bioinformatics Analysis. Front Surg. 2022; 9: 856446.

6.	Liu S, Wang W, Zhao Y, Liang K, Huang Y. Identification of Potential Key Genes for Pathogenesis and Prognosis in Prostate Cancer by Integrated Analysis of Gene Expression Profiles and the Cancer Genome Atlas. Front Oncol. 2020; 10: 809.

7.	Shan M, Xia Q, Yan D, Zhu Y, Zhang X, Zhang G, et al. Molecular analyses of prostate tumors for diagnosis of malignancy on fine-needle aspiration biopsies. Oncotarget. 2017; 8: 104761-71.

8.	Knudsen BS, Kim HL, Erho N, Shin H, Alshalalfa M, Lam LLC, et al. Application of a Clinical Whole-Transcriptome Assay for Staging and Prognosis of Prostate Cancer Diagnosed in Needle Core Biopsy Specimens. J Mol Diagn. 2016; 18: 395-406.

9.	Luca BA, Brewer DS, Edwards DR, Edwards S, Whitaker HC, Merson S, et al. DESNT: A Poor Prognosis Category of Human Prostate Cancer. Eur Urol Focus. 2018; 4: 842-50.

10.	Li C, Pang L, Jin F, Song Y, Zhou D, Song Y, et al. Integrated Network Analysis to Determine CNN1, MYL9, TAGLN, and SORBS1 as Potential Key Genes Associated with Prostate Cancer. Clin Lab. 2023; 69.

11.	Schimmelpfennig C, Rade M, Füssel S, Löffler D, Blumert C, Bertram C, et al. Characterization and evaluation of gene fusions as a measure of genetic instability and disease prognosis in prostate cancer. BMC Cancer. 2023; 23: 575.

12.	Lefort K, Ostano P, Mello-Grand M, Calpini V, Scatolini M, Farsetti A, et al. Dual tumor suppressing and promoting function of Notch1 signaling in human prostate cancer. Oncotarget. 2016; 7: 48011-26.

13.	Ta HQ, Whitworth H, Yin Y, Conaway M, Frierson HF, Jr., Campbell MJ, et al. Discovery of a novel long noncoding RNA overlapping the LCK gene that regulates prostate cancer cell growth. Mol Cancer. 2019; 18: 113.

14.	Friedrich M, Wiedemann K, Reiche K, Puppel SH, Pfeifer G, Zipfel I, et al. The Role of lncRNAs TAPIR-1 and -2 as Diagnostic Markers and Potential Therapeutic Targets in Prostate Cancer. Cancers (Basel). 2020; 12.

15.	Qiao S, Zhang W, Su Y, Jiang Y. Integrated bioinformatics analysis of IFITM1 as a prognostic biomarker and investigation of its immunological role in prostate adenocarcinoma. Front Oncol. 2022; 12: 1037535.

16.	Liu S, Zhu J, Green D, Zhong H, Long Q, Wu C, et al. Integrating Multi-Omics Data to Uncover Prostate Tissue DNA Methylation Biomarkers and Target Genes for Prostate Cancer Risk. Mol Carcinog. 2025; 64: 83-90.

17.	Varambally S, Yu J, Laxman B, Rhodes DR, Mehra R, Tomlins SA, et al. Integrative genomic and proteomic analysis of prostate cancer reveals signatures of metastatic progression. Cancer Cell. 2005; 8: 393-406.

18.	Li S, Shi L, Li F, Yao B, Chang L, Lu H, et al. Establishment of a Novel Combined Nomogram for Predicting the Risk of Progression Related to Castration Resistance in Patients With Prostate Cancer. Front Genet. 2022; 13: 823716.

19.	Ragnum HB, Vlatkovic L, Lie AK, Axcrona K, Julin CH, Frikstad KM, et al. The tumour hypoxia marker pimonidazole reflects a transcriptional programme associated with aggressive prostate cancer. Br J Cancer. 2015; 112: 382-90.

20.	Kumar A, Coleman I, Morrissey C, Zhang X, True LD, Gulati R, et al. Substantial interindividual and limited intraindividual genomic diversity among tumors from men with metastatic prostate cancer. Nat Med. 2016; 22: 369-78.

21.	Jain S, Lyons CA, Walker SM, McQuaid S, Hynes SO, Mitchell DM, et al. Validation of a Metastatic Assay using biopsies to improve risk stratification in patients with prostate cancer treated with radical radiation therapy. Ann Oncol. 2018; 29: 215-22.
